# The analytical impact of extracellular vesicles PSA on different commercial total PSA measurement methods

**DOI:** 10.11613/BM.2026.010703

**Published:** 2025-12-15

**Authors:** Ana Moreno, Amaia Sandúa, Roser Ferrer-Costa, Conxita Jacobs-Cacha, Nerea Varo, Javier Ancizu-Marckert, Jose Enrique Robles, Jose Luis Pérez Gracia, Estibaliz Alegre, Álvaro González

**Affiliations:** 1 Service of Biochemistry, Clínica Universidad de Navarra, Pamplona, Spain; 2 Laboratory of circulating biomarkers in cancer, Cancer Center Clínica Universidad de Navarra, Pamplona, Spain; 3 Clinical Biochemistry Department, Vall d’Hebron University Hospital, Barcelona, Spain; 4 Clinical Biochemistry, drug delivery and therapy Research Group, Vall d’Hebrón Research Institute (VHIR), Barcelona, Spain; 5 Department of Urology, Clínica Universidad De Navarra, Pamplona, Spain; 6 Department of Oncology, Clínica Universidad de Navarra, Pamplona, Spain; 7 IdiSNA, Navarra Institute for Health Research, Pamplona, Spain

**Keywords:** prostate-specific antigen, extracellular vesicles, immunoassays, prostate cancer, evaluation

## Abstract

**Introduction:**

Prostate-specific antigen (PSA) can circulate bound to extracellular vesicles (EVs) and its measurement (ev-PSA) can be useful in prostate cancer. Although not designed with that purpose, total PSA assays react with ev-PSA. We evaluated the analytical performance of several total PSA assays in ev-PSA quantification and the impact of ev-PSA on total PSA measurement.

**Materials and methods:**

Extracellular vesicles were isolated from 83 serum samples from prostate cancer patients by size exclusion chromatography or ultracentrifugation. PSA was quantified in serum, EVs, International Standard for PSA 17/100 from the World Health Organization (WHO IS 17/100) and exosomes from lymph node carcinoma of the prostate (LNCaP) cell line, using commercial immunoassays (Elecsys, Atellica, Immulite, Liaison and Kryptor).

**Results:**

Nanoparticle tracking analysis showed that the WHO IS 17/100 contains significantly less EVs than serum (P < 0.001). The sensitivity to detect ev-PSA followed this order: Elecsys ~ Atellica > Immulite > Liaison > Kryptor. Ev-PSA could be detected in all serum samples with Elecsys and Atellica, but not with Immulite (87.8%), Liaison (58.5%) or Kryptor (48.8%). Bland-Altman analysis showed a proportional bias in ev-PSA quantification between Elecsys and other methods. Addition of ev-PSA to serum samples caused a proportional bias in PSA measurement between Elecsys and Immulite methods, with a relationship (r^2^ = 0.99; P < 0.001) between ev-PSA and the difference in total PSA concentration between both methods.

**Conclusions:**

While ev-PSA can be measured using commercial kits, notable differences exist between methods, which could lead to potential discrepancies in serum total PSA results across various assays.

## Graphical abstract

**Figure:**
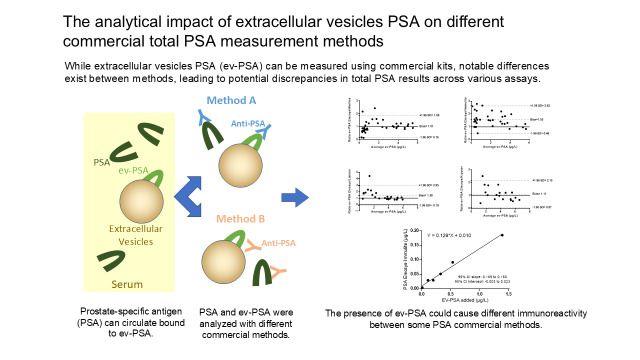

## Highlights

• PSA International Standard has less extracellular vesicles and extracellular vesicles-PSA (ev-PSA) than serum samples

• PSA detection limit may impair ev-PSA quantification by some methods

• Ev-PSA concentrations differ between methods

• Ev-PSA can cause bias in serum PSA quantification between methods

## Introduction

Prostate cancer is the most prevalent cancer in males and the second cause of cancer-related mortality (*1*). Approximately half of all males over the age of 70 will eventually be diagnosed with prostate cancer provoking high medical, psychological and economic costs for both patients and health systems. Prostate-specific antigen (PSA) plays a pivotal role in the diagnosis, prognosis, treatment selection, and follow-up of prostate cancer (*2*). Prostate-specific antigen circulates as: free PSA and PSA bound to either α1-antichymotrypsin (complexed PSA) or α2-macroglobulin (*3*). The latter is an immunologically hidden isoform, not recognized by routine analytical methods. Total PSA tests are designed to detect free PSA and complexed PSA with an equimolar response using antibodies that recognize both isoforms of the PSA molecule (*4*). These tests are calibrated with the International Standard from the World Health Organization (WHO IS) for PSA (first coded 96/670 and second coded 17/100), where the proportion of bound to free PSA is 90:10 (*5*). Despite these harmonization attempts, discrepancies between PSA concentrations quantified with different methodologies persist, thereby rendering the transfer of results challenging (*6*-*8*).

Extracellular vesicles (EVs) are small lipid membrane vesicles secreted by almost all cells into the extracellular space (*9*). Extracellular vesicles comprise exosomes (30-150 nm), originated as intraluminal vesicles within multivesicular bodies; microvesicles (100-1000 nm), formed by outward budding of the plasma membrane; and apoptotic bodies (500-2000 nm), released during programmed cell death (*9*).

Extracellular vesicles serve as carrier systems for parental cell-specific bioactive molecules, which modulate signaling pathways in recipient cells, regulating intercellular communication in a multitude of physiological and pathological processes (*10*-*12*). Active secretion of EVs is increased in cancer cells, contributing to tumor development (*13*). Present in accessible body fluids (blood, urine, semen, *etc.*), EVs are emerging as potentially clinically useful biomarkers (*13*, *14*). Although a test kit based on nucleic acid analysis in EVs has been developed to diagnose prostate cancer, the application of EVs analysis in clinical laboratories is still a challenging issue (*15*). Some of the main problems are the use of complex technologies and the lack of standardized and reproducible methods that could be transferred between laboratories (*16*, *17*).

Recent studies have demonstrated that PSA also circulates bound to EVs (ev-PSA) (*18*). Additionally, the proportion of ev-PSA is increased when serum total PSA is lower than 4 μg/L, differing significantly between prostate cancer patients and healthy controls or patients with benign hyperplasia (*19*, *20*). Moreover, some commercial PSA kits, despite not being designed to react with this molecular form of PSA, do in fact recognize it (*19*). Total PSA assays use antibodies that may react differently, or even not react, with ev-PSA. Due to these reactivity differences, the presence of ev-PSA might cause a bias in the quantification of PSA, which can be quite relevant in some patients, as ev-PSA might account for more than 30% of serum total PSA (*19*).

Our aim was to evaluate the analytical performance of total PSA commercial methods in the quantification of ev-PSA, and to assess the impact of that ev-PSA on serum total PSA measurement.

## Materials and methods

### Subjects

Blood samples were obtained from 83 prostate cancer patients, who attended the Medical Oncology Department of our institution between 2023 and 2024. Storage time was less than two years, which does not affect PSA stability (*21*). Samples were collected in 5 mL or 10 mL Vacutainer serum collection tubes (Becton Dickinson, Sunnyvale, USA). After clot formation, tubes were centrifuged at 2000xg for 10 min and serum samples were then aliquoted and stored at - 80°C in the collection C.0003132; 14/04/2014, of the National Biobank Registry. The study was approved by the Local Ethic Committee (2022.087), and all volunteers gave written informed consent for the study.

Commercial exosomes derived from the Lymph node carcinoma of the prostate (LNCaP) cell line were purchased from HansaBioMed Life Sciences (Tallinn, Estonia). The WHO IS PSA coded 17/100, was purchased from the National Institute for Biological Standards and Control (NISBC, South Mimms, UK) and reconstituted according to the manufacturer´s instructions. This standard replaces the exhausted stock 1st WHO IS coded 96/670.

### Methods

#### Extracellular vesicles isolation

**Figure 1:**
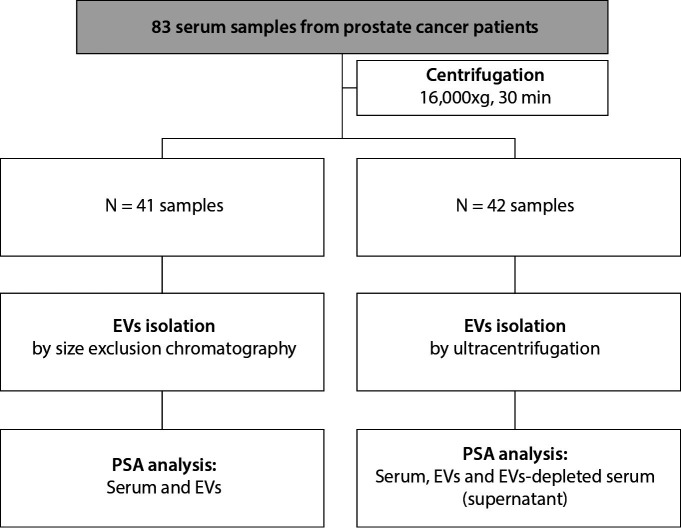
Flowchart showing the distribution of 83 serum samples from prostate cancer patients across the different analyses performed. EVs - extracellular vesicles. PSA - prostate specific antigen.

Serum samples from 41 patients, randomly selected, were initially centrifuged at 16,000xg for 30 minutes to eliminate any residual cellular debris (Figure 1). Extracellular vesicles were then isolated using size exclusion chromatography (SEC) with the Exo-spin midi kit columns (Cell Guidance System, Cambridge, UK), in accordance with the manufacturer’s instructions.

To obtain both EVs-depleted serum and EVs from the same sample, 42 randomly selected serum samples were centrifuged at 16,000xg for 30 min and subsequently subjected to ultracentrifugation at 100,000xg for 90 min in a Hitachi CS150NX micro ultracentrifuge (Hitachi Koki Co., Tokyo, Japan). After centrifugation, the EVs-depleted serum (supernatant, sn) was carefully removed and the pellet containing the EVs was resuspended in female serum previously deprived of EVs by ultracentrifugation.

Additionally, the WHO IS 17/100 diluted (1:4) in phosphate buffered saline (PBS) was ultracentrifuged and resuspended in PBS.

#### Nanoparticle tracking analysis

The presence of EVs was analyzed in the WHO IS 17/100 and in a serum sample with a PSA concentration of 100.9 µg/L, used as a positive control, by Nanoparticle tracking analysis with a NanoSight LM20 (Malvern Panalytical, Malvern, UK). Particle size and concentration were determined and reported as nm and particles/mL, respectively.

#### Prostate specific antigen analysis

The concentrations of total PSA in serum (s-PSA), in serum-derived EVs (ev-PSA) and supernatants (sn-PSA) were measured using total PSA commercial assay kits with their respective analyzers: Elecsys (Cobas e602, Roche Diagnostics, Basel, Switzerland), Atellica (Atellica IM 1600, Siemens Healthineers, Erlangen, Germany), Immulite (Immulite 2000 XPi, Siemens Healthineers, Erlangen, Germany), Kryptor (Brahms Kryptor Compact Plus, Thermo Fisher, Waltham, USA), and Liaison (Liaison XL, Diasorin, Saluggia, Italy). All these methods are calibrated against the WHO IS PSA 96/670, but differ in the capture and tracer antibodies against different PSA epitopes and their declared limits of quantification (Table 1) (*4*).

**Table 1:** Rate of ev-PSA detection and ev-PSA concentrations in 41 samples from prostate cancer patients quantified by different immunoassays

**Assay**	**Declared LOQ (µg/L)**	**% of samples with detected ev-PSA**	**ev-PSA** **(µg/L)**
Elecsys	0.014	100	1.06 (0.43-3.68)
Atellica	0.02	100	0.92 (0.55-3.66)
Immulite	0.04	87.8	1.24 (0.34-2.68)
Kryptor	0.27	48.8	3.59 (1.85-5.91)
Liaison	0.09	58.5	3.44 (0.77-4.82)
ev-PSA - prostate specific antigen quantified in extracellular vesicles. LOQ - limit of quantification. ev-PSA is presented as median and interquartile range.			

#### Linearity and detection limit analysis

Samples were prepared by serial dilutions of LNCaP exosomes using women´s EVs free serum supernatant. Linearity analysis was performed following the CLSI EP06 recommendations (*22*). The detection limit study was performed following the Spanish Society of Laboratory Medicine (SEQC-ML) recommendations (*23*).

#### Serum spiking with ev-PSA

A pool of women serum (400 µL) was spiked-in with 100 µL of five different concentrations of ev-PSA (0.529 to 6.93 µg/L) diluted in PBS or with 100 µL of PBS alone (Table 2). Prostate specific antigen was then quantified in duplicate using the Elecsys and Immulite assays.

**Table 2:** Results from the spiking experiment with ev-PSA

**Samples**	**Spiking evPSA (µg/L)**	**Dilution**	**Expected evPSA (µg/L)**	**PSA Elecsys** **(µg/L)**	**PSA Immulite** **(µg/L)**	**Δ PSA (Elecsys - Immulite)**
1	0.529	1:5	0.106	0.106	0.077	0.029
2	0.979	1:5	0.196	0.189	0.160	0.029
3	1.61	1:5	0.322	0.322	0.271	0.051
4	2.66	1:5	0.532	0.535	0.444	0.091
5	6.93	1:5	1.386	1.380	1.195	0.185
A pool of women serum was spiked-in with five different concentrations of ev-PSA and total PSA was quantified in duplicate by Elecsys and Immulite assays. The PSA concentrations correspond to duplicates means. Δ PSA corresponds to the difference between PSA measured by Elecsys and PSA measured by Immulite. ev-PSA - prostate specific antigen quantified in extracellular vesicles. PSA - prostate specific antigen.						

### Statistical analysis

Sample size (N = 34) for each group was calculated based on an alpha of 0.05, a power of 0.8, and an expected effect size of 0.5. D’Agostino-Pearson normality test was used to study data distribution. Data was represented as median and interquartile range. Correlation was studied using Spearman correlation coefficient (r). When comparing the methods, ev-PSA results with the other immunoassays were referred to Elecsys results, as this was the method employed in a previous publication (*19*). For comparisons, Friedman test and Dunn’s multiple comparisons test were used. Regression analysis was performed with the Deming test and agreement between methods was assessed with Bland-Altman test (*24*). A two-tailed P-value of < 0.05 was considered statistically significant. Statistical analysis was performed using GraphPad Prism version 10.3 (GraphPad Software, Boston, USA).

## Results

### Analysis of ev-PSA in the WHO International Standard 17/100 for total PSA

We observed that the mean particle concentration in the ultracentrifuged serum sample was 6.6 x10^8^ ± 5.9 x10^7^ particles/mL (101.5 ± 9.8 particles/frame) with a mean size of 115 ± 53 nm, and ev-PSA represented 0.66% of total PSA. However, the mean particle concentration in the WHO IS 17/100 was more than 10 times lower, 4.7 x10^7^ ± 3.7 x10^6^ particles/mL (7.8 ± 0.6 particles/frame) with a mean size of 135 ± 39 nm, and ev-PSA was only 0.2% of total PSA (P < 0.001).

### Study of the ev-PSA reactivity to immunochemical methods

We previously observed that commercial exosomes from the LNCaP prostate cell line showed a total PSA concentration of 35.3 μg/g of exosomes using the Elecsys total PSA commercial assay (*19*). When serial dilutions were performed, all immunoassays showed a good linearity in the measuring range (dilution 1 corresponds to 2.48 µg/L of ev-PSA measured with Elecsys, Supplemental Figure 1), with a r^2^ > 0.97, and the 95% confidence intervals of the X- Y- intercepts containing the 0 value. The coefficient of variation at the concentrations of 0.2 and 0.1 µg/L was lower than 5% with all methods analyzed. However, the assays showed a very different analytical sensitivity, being higher for Elecsys and Atellica, which could detect a relative concentration of 0.016. Meanwhile, Immulite did not detect relative concentrations lower than 0.06, and Kryptor and Liaison lower than 0.16.

As the Elecsys method showed the best analytical sensitivity, we then studied its detection and quantification limits for ev-PSA, which were 0.01 µg/L and 0.02 µg/L, respectively. We replicated the study with the Immulite assay, which in comparison had higher detection and quantification limits: 0.04 µg/L and 0.11 µg/L, respectively. In both cases, the quantification limits were similar to those reported by the manufacturer for serum total PSA.

### Circulating ev-PSA evaluation with different commercial immunoassays

**Figure 2:**
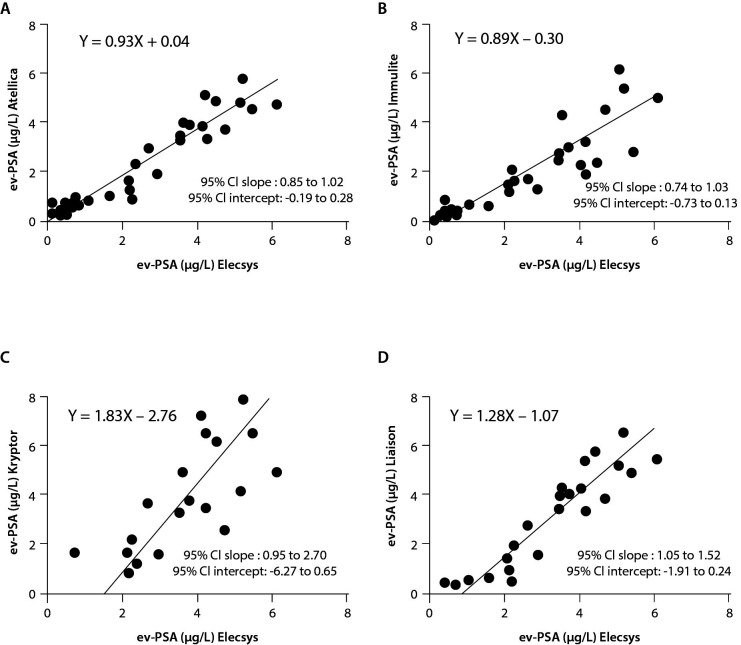
Deming regression analysis of ev-PSA measured with: (A) Elecsys and Atellica, (B) Immulite, (C) Kryptor, or (D) Liaison immunoassays.

We analyzed 41 samples with a median s-PSA concentration of 25.74 µg/L (12.16-72.32 µg/L), according to the Elecsys method (Figure 1). After EVs isolation by SEC, ev-PSA was detected in 100% of the samples when analyzed with the Elecsys and Atellica immunoassays but not with the other immunoassays (Table 1). In those samples (N = 20) where ev-PSA was measurable by all five methods, ev-PSA concentrations were significantly lower when measured with Immulite compared to Elecsys (P *=* 0.014) and Kryptor (P = 0.019) (Supplemental Figure 2). Concerning ev-PSA, Deming regression analysis showed that Atellica and Immulite immunoassays demonstrated the greatest concordance with the Elecsys immunoassay for ev-PSA. In contrast, Kryptor and Liaison significantly overestimated ev-PSA concentrations, with Kryptor showing the greatest deviation (Figure 2).

**Figure 3:**
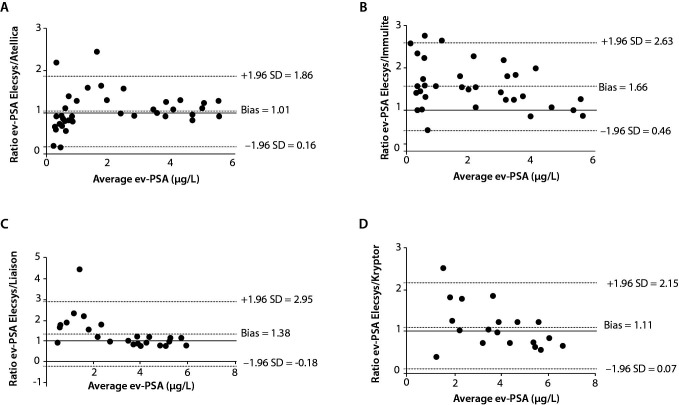
Bland-Altman plots of the ev-PSA ratio between (A) Elecsys and Atellica, (B) Immulite, (C) Liaison and (D) Kryptor assays against the mean value of the two assays. Dotted lines indicate the bias between the methods and the limits of agreement.

We also assessed the agreement between Elecsys and the other methods to measure ev-PSA concentrations with Bland-Altman plots (Figure 3). We observed that the Elecsys assay showed a high agreement with Atellica assay, but there was a proportional bias compared to the other assays. For ev-PSA concentrations below 3 µg/L, the Elecsys assay reported higher concentrations than the Immulite, Kryptor or Liaison assays, and for ev-PSA concentrations below 1 µg/L, lower concentrations than the Atellica assay.

### Comparison of the reactivity of ev-PSA and soluble total PSA

**Figure 4:**
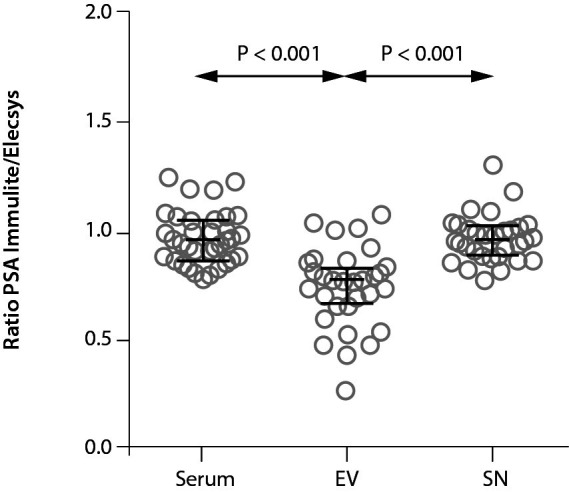
Comparison of the Immulite/Elecsys ratio of PSA concentrations measured in serum, extracellular vesicles (EV), and EV-free serum supernatant (SN). Lines and whiskers represent median and interquartile range. PSA - prostate specific antigen.

To assess whether the immunoreactivity differences were more relevant for ev-PSA than for the soluble forms present in the supernatant, we compared PSA concentrations in EVs and in EVs-free serum supernatant (sn-PSA) obtained by ultracentrifugation from 42 patients. Based on previous results, we focused on Elecsys and Immulite PSA methods (Figure 4 and Supplemental Table 1). Immulite/Elecsys ratio of sn-PSA was 0.96 (0.89 - 1.02), similar to that of serum (0.96 (0.86 - 1.05); P *=* 0.057), while in EVs the ratio decreased to 0.77 (0.66 - 0.83; P *<* 0.001). These results showed that ev-PSA exhibited different immunoreactivity compared to soluble sn-PSA when measured with Elecsys and with Immulite assays.

### Impact of ev-PSA on discrepancies in total PSA results across immunoassays

**Figure 5:**
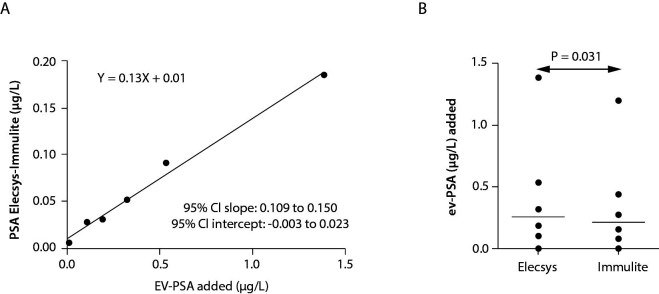
(A) Relationship between the ev-PSA added and the difference between PSA measured with Elecsys and Immulite. (B) Comparison of the concentration measured with Elecsys and Immulite for different amounts of ev-PSA added to a woman serum sample. Horizontal lines represent median. PSA - prostate specific antigen.

The spiking experiment performed to study the impact of the different ev-PSA reactivity on serum total PSA measurement showed a relationship between the concentration of the added ev-PSA and the difference in total PSA concentration between Elecsys and Immulite (r^2^ = 0.99; Table 2 and Figure 5A). Furthermore, we observed that Elecsys produced significantly higher results than Immulite (P = 0.031; Figure 5B), with a mean bias of 18% ± 6%. To address that the difference was not due to matrix effect, we also measured samples to which only PBS was added and there was no significant difference between the two methods (P > 0.999).

Regarding the other methods, we analyzed the differences between s-PSA results related to ev-PSA measured in EVs obtained by SEC. We observed a significant correlation between the difference of s-PSA between Kryptor or Liaison and Elecsys in relation to ev-PSA measured with Elecsys (r = 0.59; P *<* 0.001) and (r = 0.49*;* P *=* 0.001) respectively. Of note, there was a poor but significant correlation between ev-PSA measured with Atellica and the differences in serum PSA between the two Siemens methods, Atellica and Immulite (r = 0.37; P *=* 0.019). This correlation was also observed when we analyzed the ev-PSA in EVs obtained by ultracentrifugation, which confirmed that this effect was not due to EVs extraction method.

## Discussion

In this work, we evaluated the quantification of ev-PSA using different commercial methods designed for the analysis of serum PSA. These commercial kits were selected because they are routinely used in clinical laboratories, their antibodies reactivity has been described, and their harmonization has been analyzed in most of them (*4*, *6*, *7*). The possibility of using these PSA assays to determine the concentration of PSA in EVs was already established (*19*). However, these commercial methods were not designed to measure this circulating form of PSA, and their epitopes can be unexposed or hidden within these particles. Moreover, the methods analyzed here use antibodies that recognize different epitopes present in the PSA molecule and have different affinities (*4*). We observed that all of them reacted with ev-PSA, which means that the recognized epitopes are exposed to the antibodies. This suggests that ev-PSA is located at the surface of the EVs, either integrated or, more likely, associated with the membrane (*25*).

The concentration of ev-PSA is lower than the soluble circulating isoforms and, in most cases, close to the quantification limit (*19*). We observed that all the methods showed linearity in the response below 2.5 µg/L, which is the range of the ev-PSA concentration usually present in serum. The sensitivity to ev-PSA changed noticeably between the immunochemical methods studied, with Elecsys and Atellica being the most sensitive of the assays analyzed here. It is interesting to note that the sensitivity differs between the two Siemens methods, being lower in the Immulite assay than in the Atellica method. However, all of them presented a good reproducibility.

Most commercial PSA kits are calibrated using the WHO IS 96/670 for PSA, which allows equimolar reactivity with total and free PSA, thereby reducing interlaboratory coefficients of variation in the measurement of the major serum PSA isoforms (*5*). However, discordances among laboratories for serum PSA concentrations are approximately 15% (*26*). At the time this standard was developed, ev-PSA had not been described (*27*). This standard is obtained from seminal fluid, which is rich in EVs (*28*), but we have observed that it contains very low concentrations of EVs and ev-PSA. The low levels of ev-PSA in the WHO IS 17/100 and the use of antibodies not designed to recognize it in an equimolar way may also cause a bias in the comparison of the s-PSA reactivity between different methods. This can occur especially when the proportion of ev-PSA is high, which is more common when s-PSA is less than 4 µg/L (*19*). This bias can be quite relevant as in some patients, ev-PSA might account for more than 30% of serum total PSA, and even higher than the percentage of free PSA.

We have observed that ev-PSA reacts differently depending on the commercial method used. Hence, there was an important bias when comparing Immulite, Kryptor and Liaison with Elecsys assay. However, as expected, there was a strong agreement between these assays when analyzing s-PSA (*7*). Also, while the Elecsys and Atellica assays determined ev-PSA concentrations in all samples (some very close to the limit of quantification), Liaison and Kryptor failed to quantify most of them. The difference in the recognition of the soluble form of PSA and ev-PSA was also demonstrated with the higher correlation and agreement between methods for s-PSA and sn-PSA, compared to ev-PSA. This is especially relevant in the case of Kryptor assay, where although the limit of detection was higher than that of Elecsys and could not detect ev-PSA in most samples, there was an overestimation of ev-PSA concentration in the measured range. This could be due to the different epitopes recognized by the monoclonal antibodies that react distinctly with ev-PSA (*4*). Ferraro *et al.* showed a general tendency of the Roche assays to overestimate and of the Siemens assays to underestimate serum PSA concentrations (*6*). The observed overestimation also holds true for ev-PSA and even to a greater degree, but with a difference between Atellica and Immulite. It is interesting to note that the difference in serum total PSA between these two Siemens Healthineers methods correlates with the concentration of ev-PSA in the samples. Importantly, those differences in ev-PSA reactivity affecting PSA quantification, have been observed using two EVs isolation methods (SEC and ultracentrifugation), which suggests that our observations were not influenced by the EVs purification method (*17*). Considering all this, we can conclude that the different immunoreactivity of ev-PSA might cause a bias between serum PSA commercial methods.

Despite the efforts made for the harmonization of PSA measurement, it was observed that the bias was not within the analytical performance specifications (± 10.6%) based on biological variation, as previously described by the European Biological Variation Study (*29*). The type of antibody can contribute to the bias between different methods. However, if the concentration of ev-PSA is high in a serum sample, it could produce different serum total PSA results with different methods due to the distinct reactivity of ev-PSA, as shown here, and this could have implications for the transfer of serum results between laboratories that use different methods. The effect of ev-PSA is relevant at concentrations lower than 4 µg/L, where the percentage of this form can be higher than 10% and even reach 40% of total PSA (*19*). Probably, at high PSA concentrations, where the proportion of ev-PSA is usually much lower, the effect could be negligible. For this reason, in the standardization of the methodology, commercial kits should consider the presence of ev-PSA and design antibodies that also react equimolarly with ev-PSA. Further studies would be needed to address this relevant point.

Since observed changes in EVs should undoubtedly be attributed to changes in the clinical status of the patient and not to differences in the isolation and/or analysis process, the use of EVs as a liquid biopsy in clinical practice has been hampered by the lack of standardization of techniques for EVs isolation and subsequent analysis (*9*, *17*). Furthermore, this lack of standardization also limits the reproducibility of the measurement, making it difficult to compare and share results between different research groups (*18*, *30*). Many of the test protocols usually used are not commercially available and lack reference materials and experimental controls to be reliably used for standardization of experiments between laboratories. We have previously shown that using the Elecsys total PSA assay, the ev-PSA/s-PSA ratio can differentiate prostate cancer patients from both benign prostatic hyperplasia patients and healthy controls (*19*). For this reason, the use of standardized commercial kits could facilitate comparison between laboratories. However, a methodological analysis of commercial kits for use with EVs, as shown here, is necessary to facilitate interlaboratory comparisons and multicenter studies.

The reactivity of the method used against ev-PSA can lead to over- or underestimation of total PSA depending on the assay used. At s-PSA concentrations lower than 4 µg/L, where the ratio ev-PSA/s-PSA is higher, is probably where ev-PSA has a more significant effect on the bias between methods (*19*). This bias can cause relevant clinical implications, as the proportion of ev-PSA to total PSA is higher in patients with benign prostatic hyperplasia compared to cancer patients (*19*). In addition, it could affect patient follow-up with total PSA when ev-PSA concentrations change during the course of the disease. In addition, it could explain the discrepancy in total PSA results obtained with certain measurement systems in specific patient subsets, impairing the interchangeability of methods.

Our study has two main limitations. First, we focused on technical performance rather than clinical performance. However, our results suggest that, at least, the ev-PSA could influence the use of different commercial kits for the monitoring of cancer patients. Second, we evaluated only a subset of commercially available PSA assays. Further studies would be needed with more commercial methods and performing an in-depth analysis of the s-PSA range where the effect of ev-PSA would have more impact. In summary, here we show that ev-PSA can be measured using commercial kits such as Elecsys, Atellica, Immulite, Kryptor and Liaison, not designed for this form of PSA. It should be considered that ev-PSA measurement is method-dependent and that the detection limit for ev-PSA could impair its detection and quantification (*8*). Finally, ev-PSA can be a source of discrepancies in the routine serum total PSA results obtained with different commercial PSA assays and should be considered when transferring results between laboratories.

## Data Availability

The data generated and analyzed in the presented study are available from the corresponding author on request.
